# Novel Conjugative Transferable Multiple Drug Resistance Plasmid pAQU1 from *Photobacterium damselae* subsp. *damselae* Isolated from Marine Aquaculture Environment

**DOI:** 10.1264/jsme2.ME11338

**Published:** 2012-03-23

**Authors:** Lisa Nonaka, Fumito Maruyama, Manabu Miyamoto, Masatoshi Miyakoshi, Ken Kurokawa, Michiaki Masuda

**Affiliations:** 1 Department of Microbiology, Dokkyo Medical University School of Medicine, Mibu, Tochigi, 321–0293, Japan; 2 Section of Bacterial Pathogenesis, Section of Microbial Genomics and Ecology, Tokyo Medical and Dental University, Graduate School of Medical and Dental Science, Bunkyo-ku, Tokyo, 113–8510, Japan; 3 Department of Environmental Life Sciences, Graduate School of Life Sciences, Tohoku University, Katahira 2–1–1, Sendai 980–8577, Japan; 4 Department of Biological Information, Tokyo Institute of Technology, Graduate School of Bioscience and Biotechnology, Yokohama, Kanagawa 226–8501, Japan

**Keywords:** multi-drug resistance, conjugative plasmid, aquaculture environment

## Abstract

The emergence of drug-resistant bacteria is a severe problem in aquaculture. The ability of drug resistance genes to transfer from a bacterial cell to another is thought to be responsible for the wide dissemination of these genes in the aquaculture environment; however, little is known about the gene transfer mechanisms in marine bacteria. In this study, we show that a tetracycline-resistant strain of *Photobacterium damselae* subsp. *damselae*, isolated from seawater at a coastal aquaculture site in Japan, harbors a novel multiple drug resistance plasmid. This plasmid named pAQU1 can be transferred to *Escherichia coli* by conjugation. Nucleotide sequencing showed that the plasmid was 204,052 base pairs and contained 235 predicted coding sequences. Annotation showed that pAQU1 did not have known *repA*, suggesting a new replicon, and contained seven drug resistance genes: *bla*_CARB-9_-like, *floR*, *mph*(A)-like, *mef*(A)-like, *sul2*, *tet*(M) and *tet*(B). The plasmid has a complete set of genes encoding the apparatus for the type IV secretion system with a unique duplication of *traA*. Phylogenetic analysis of the deduced amino acid sequence of relaxase encoded by *traI* in pAQU1 demonstrated that the conjugative transfer system of the plasmid belongs to MOB_H12_, a sub-group of the MOB_H_ plasmid family, closely related to the IncA/C type of plasmids and SXT/R391 widely distributed among species of Enterobacteriaceae and Vibrionaceae. Our data suggest that conjugative transfer is involved in horizontal gene transfer among marine bacteria and provide useful insights into the molecular basis for the dissemination of drug resistance genes among bacteria in the aquaculture environment.

The emergence and spread of drug-resistant bacteria is a global public health problem. This problem is impacted by both human and non-human use of antimicrobial agents. For example, in aquaculture, antimicrobial agents are used prophylactically to protect cultured animals from infectious diseases and avoid economic losses. Use of antimicrobial agents leads to selection for drug-resistant bacteria (6).

The occurrence of tetracycline-resistant bacteria in the sediment and seawater of an aquaculture site significantly increased after using oxytetracycline (38). Additionally, prior acquisition of drug resistance gene(s) by bacteria is a major factor for the generation and propagation of drug-resistant bacteria. Previously, the common tetracycline resistance gene, *tet*(M), was found in several distantly related species of bacteria isolated from fish at an aquaculture site (23), as well as from the sediment and seawater at the same site (38); therefore, gene transfer among different species of bacteria appears to be involved in the acquisition of drug resistance gene(s) in the aquaculture setting. Previously, we found that the majority of the *tet*(M)-positive isolates belonged to closely related genera, *Vibrio* and *Photobacterium*, suggesting that bacteria from this group might serve as an important reservoir for *tet*(M) in the aquaculture environment (38), and *tet*(M) in some bacteria could be transferred to *Escherichia coli* and confer drug resistance (36). Among the bacteria from this group, *P. damselae* subsp. *damselae* is an indigenous marine bacterium (1, 46) known to be pathogenic in both fish and humans. In fish, it causes septicemia in a broad range of species, *e.g.*, damselfish and rainbow trout (29, 40, 41). In humans, exposure to seawater or fish contaminated with *P. damselae* may cause fatal necrotizing fasciitis (2, 12, 17, 34, 52, 59). Some clinical isolates of *P. damselae* subsp. *damselae* are drug-resistant, leading to obstacles for chemotherapy (59); therefore, drug resistance in aquatic bacteria may possibly pose a risk for human health either directly or indirectly. To evaluate this risk, determining the characteristics of drug resistance genes carried by *P. damselae* subsp. *damselae* and the transfer mechanism of these genes to human enteric bacteria is required.

Here we determined the molecular basis for tetracycline resistance in a strain of *Photobacterium damselae* subsp. *damselae* isolated from seawater at a coastal aquaculture site in Japan. The results show that the strain has a transferable plasmid named pAQU1, of approximately 200 kilo base pairs (kbp). Nucleotide sequencing showed that the plasmid contains seven drug resistance genes and a complete set of genes encoding the apparatus for the type IV secretion system (T4SS) with a unique duplication of *traA*.

From the deduced amino acid sequence of the relaxase encoded by *traI*, pAQU1 was classified as a novel member of the MOB_H_ plasmid family in sub-group MOB_H12_. The phylogenetic relationship of this plasmid compared to other known groups of plasmids and integrative conjugative elements widely distributed among the species of Enterobacteriaceae and Vibrionaceae is discussed. Many reports have shown the prevalence of drug resistance among fish pathogens (6, 24, 33) and aquaculture environmental bacteria (15, 38), whereas there is little information concerning the mechanism of their emergence. Our data provide useful insights into the molecular basis for the dissemination of drug resistance genes among bacteria in the aquaculture environment and their clinical impact.

## Materials and Methods

### Bacterial strains

*P. damselae* subsp. *damselae* strain 04Ya311 was isolated from seawater at a coastal aquaculture site in Japan and was used as the conjugation donor (38). Its identification was confirmed using a full-length 16S rRNA gene sequence and PCR detection of the *ureC* gene (39). *E. coli* K-12 strain W3110 (whole genome sequence accession no. AP000091; 19) was obtained from the National BioResource Project (National Institute of Genetics, Japan) and used as the conjugation recipient. Rifampicin-resistant W3110Rif^r^, used as the recipient in the alternative transfer experiment, was artificially obtained by induction. The *P. damselae* subsp. *damselae* donor strain was cultured in brain heart infusion medium (BHI; BD, Franklin Lakes, NJ) with 2% NaCl and 20 μg mL^−1^ tetracycline at 25°C. The transconjugant and recipient *E. coli* strains were cultured at 37°C in Luria Bertani medium (LB; BD) with and without 20 μg mL^−1^ tetracycline, respectively.

### Filter mating

The donor strain was cultured to the mid-log phase in BHI with 2% (w/v) NaCl and 20 μg mL^−1^ tetracycline at 25°C. The recipient strain was cultured to the mid-log phase in LB at 37°C. Filter mating was performed on LB agar plates at 25°C without antibiotic selection for 12 h; and the transconjugants were selected as described previously (36). To see the transferability of pAQU1 from these transconjugants to an alternative recipient, two transconjugants (TJW2 and TJW13) were used as donors and W3110Rif^r^ was used as the recipient. Filter mating was performed on LB agar plates at 37°C for 6 h; colonies that grew on the LB plates containing 20 μg mL^−1^ tetracycline and 100 μg mL^−1^ rifampicin at 37°C were selected and transfer of *tet*(M) was checked by PCR (38).

### Antimicrobial susceptibility test

Minimal inhibitory concentrations (MICs) of *E. coli* were determined using the broth dilution method according to the NCCLS guidelines (35). For *P. damselae* subsp. *damselae*, we used the same method with modifications of the salt concentration to 2% NaCl, incubation temperature (25°C) and culture period (48 h) appropriate for this microorganism (26).

### Pulsed field gel electrophoresis (PFGE) and hybridization

The total DNA from the donor and recipient strains was fractionated using PFGE according to the S1 nuclease method developed for plasmids larger than 100 kbp (3), and transferred onto a nylon membrane (GE Healthcare UK Ltd., Buckinghamshire, UK). The plasmid DNA was detected using Southern blot hybridization (50). A 637 base pairs (bp) PCR product of *tet*(M) was labeled with digoxigenin using a PCR DIG probe synthesis kit (Roche Diagnostics, Mannheim, Germany), and used as the hybridization probe.

### Sequencing and annotation

Total DNA containing both of the genomic and plasmid DNAs was extracted from a selected transconjugant using the QuickGene DNA tissue kit (Fujifilm, Tokyo, Japan) according to the manufacturer’s protocol, and the purified DNA was sent to Takara Bio (Ohtsu, Japan) for sequencing. The whole sequence of the total DNA was determined by the pyro-sequencing method with a Genome Sequence FLX system using titanium chemistry (Roche Diagnostics). Approximately 5 μg of the total DNA was used for the standard Roche 454 shotgun sequencing method, and a set of data having 628,489 sequences with an average length of 367 nucleotides was generated. The data were estimated to encompass 25 times the entire DNA sequence. From the data set, the genomic sequence of *E. coli* W3110 was subtracted leaving the plasmid-derived sequences. The plasmid-derived sequences were then assembled *de novo* using the GS assembler (Newbler 2.0; Roche Diagnostics), and three sets of assembled contigs were generated. To fill the gaps between the contigs, PCR was performed using the total DNA template with LA Taq polymerase (Takara Bio), and with primers corresponding to the sequences close to the terminal ends of each contig. The nucleotide sequences of the PCR products were determined using the dideoxy chain termination method with a Big Dye terminator V 3.1 cycle sequencing kit (Life Technologies Corporation, Carlsbad, CA). Fidelity of the final assembly of the circular plasmid was confirmed using the scanning PCR method with 26 sets of primers designed with web-based software GenoFrag (60). The primers were optimized for long-range PCR to amplify the 26 segments covering the entire length of the plasmid. Preliminary identification of protein coding regions was performed using the MetaGeneAnnotator (37). The data were compared using BLAST for sequences in the NCBI non-redundant protein database for length, identity, and coverage; the final annotation was manually performed using the In Silico Molecular Cloning Genomics Edition (In Silico Biology, Yokohama, Japan).

### Phylogenetic analysis

A BLAST search was performed using the amino acid sequence of pAQU1-encoded relaxase (TraI) as the query sequence. The TraI sequence was aligned using CLUSTAL W and compared with those of 24 other known TraI in the MOB_H_ family (16) (Table S1). Twenty-one highly conserved regions within the respective TraI sequences were joined using trimAL (8), generating 25 sets of approximately 450 amino acid sequences (Fig. S1). Finally, we used the neighbor-joining (NJ) method with 1,000 bootstrap values, and similarities were compared using Molecular Evolutionary Genetics Analysis 5 (56).

### Nucleotide sequence accession number

The entire sequence of pAQU1 is deposited in GenBank/DDBJ/EMBL databases under accession no. AB571865.

## Results and Discussion

### Transfer and detection of a 200-kbp conjugative plasmid carrying tet(M)

**Fig. 1:**
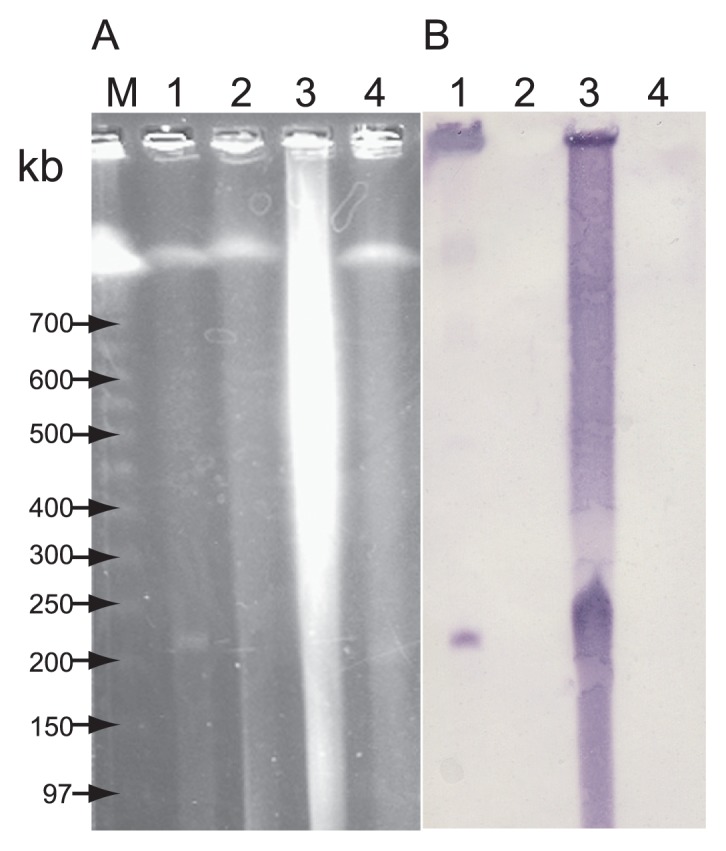
Detection of plasmid pAQU1 in the donor and a representative transconjugant using PFGE (A) and Southern hybridization with the *tet*(M) probe (B). Lanes: M, DNA size standard (lambda ladder); 1, representative transconjugant TJ311W2; 2, *E. coli* W3110; 3, *P. damselae* subsp. *damselae* 04Ya311; and 4, negative control strain of *P. damselae* subsp. *damselae* JCM8967. About 20 ng of DNA was loaded in each lane.

A previous study showed *tet*(M) was transferred from *P. damselae* subsp. *damselae* 04Ya311 to *E. coli* using conjugation with a transfer rate of (6.62±1.61)×10^−3^(36); therefore, filter mating experiments were performed to obtain transconjugants. To examine the location of the *tet*(M) gene in the *P. damselae* subsp. *damselae* 04Ya311 genome, we performed PFGE and hybridization analysis using a *tet*(M) probe. The PFGE showed that an extra-chromosomal DNA of ~200-kbp was present in *P. damselae* subsp. *damselae* 04Ya311, and the position of the *tet*(M) hybridization signal corresponded to this extra-chromosomal DNA (Fig. 1B).

PFGE and subsequent hybridization showed that extra-chromosomal DNA of ~200-kbp was present in the tetracycline-resistant transconjugant *E. coli*; but not in the recipient *E. coli* W3110 control (Fig. 1A, B). Twenty transconjugants were generated using independent filter mating experiments and were similarly examined, yielding the same data. Therefore, *tet*(M) appeared to be carried by a plasmid whose size was ~200-kbp, unlike our previous report (36). In our previous study (36), we failed to detect this 200-kbp plasmid because of the use of regular agarose gel electrophoresis, which cannot separate DNA fragments lager than approximately 20-kbp, instead of PFGE.

### General features of the pAQU1 sequence

**Fig. 2:**
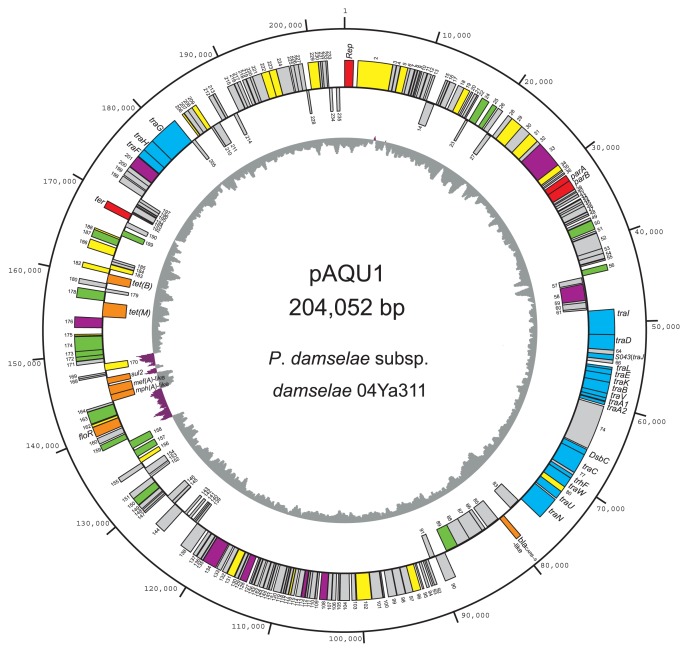
Circular map of pAQU1. CDSs outside and inside of the circle are coded clockwise and counter-clockwise, respectively. Putative functions of the products of the CDSs are indicated in color: red, replication, partition and termination; purple, DNA processing; blue, conjugative transfer; green, transposition or integration; orange, antibiotic resistance; yellow, other functions; and gray, unknown functions. The third circle indicates GC content where purple shows upper GC value above the center line and gray shows lower GC value below the center line.

To characterize the 200-kbp plasmid bearing *tet*(M), the nucleotide sequence of the total DNA extracted from the transconjugant *E. coli* was determined using the pyro-sequencing method. Because the entire sequence of *E. coli* W3110 was known, its sequence was subtracted from the whole sequence data. The three contigs after subtraction were shown to be continuous by using PCR, and further gap sequencing revealed the complete sequence of a circular plasmid, which was originally transferred from *P. damselae* subsp. *damselae*. The plasmid was named pAQU1; it contained 204,052 bp and 235 predicted coding sequences (CDSs). The gene organization and G+C contents of the respective CDSs are summarized in Fig. 2. The CDSs were asymmetrically distributed: 182 CDSs were oriented clockwise, whereas only 53 CDSs were oriented counterclockwise. When a BLAST search was performed using the deduced amino acid sequences of the CDSs, related proteins of known functions were found for 78 out of the 235 CDSs. Based on their predicted function, these CDSs were classified into eight groups (Table 1). For the other 157 CDSs, similarity to other proteins with known functions was not found (Table 1). The average G+C content of pAQU1 was 43.2%; however, there was a region of notably higher G+C contents at positions 140,000 to 150,000 (Fig. 2) that contained transposons and the *floR* gene (see “Drug resistance gene” section).

### Genes potentially involved in replication of pAQU1

Principally, plasmid-encoded Rep proteins have 3′ → 5′ helicase activity and initiate plasmid replication through binding to specific sequences in the replication origin and facilitating the interaction of host proteins with the origin (55). The deduced amino acid sequence of CDS 001 showed 33% (Table 1) identity to the putative replication protein (RepA) from *Vibrio anguillarum* and 24% identity to the RepA protein of the well-characterized plasmid RSF1010 from *E. coli*(31). Thus, although pAQU1 appears to have a putative Rep protein, its sequence similarity with known RepA is low. Moreover, sequences similar to the primer sets conventionally used for PCR-based replicon typing (20) were not found to be effective for pAQU1. This suggests that pAQU1 contains a novel replicon capable of replicating in both *P. damselae* subsp. *damselae* and *E. coli*. A previous report showed that unknown types of plasmids are distributed among marine environmental bacteria (11, 54). So far, little is known about the diversity of marine plasmids, although they seem to play an important role in the dissemination of resistance genes in the marine environment (15).

**Fig. 3:**
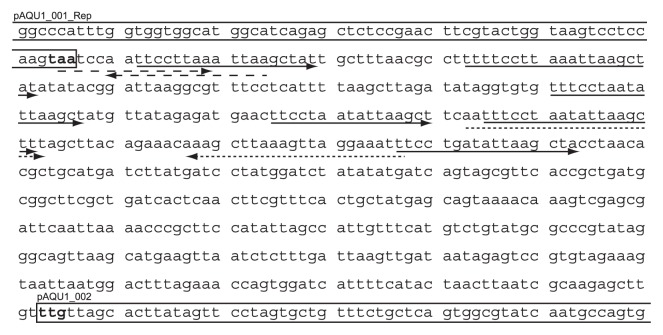
DNA sequence of the potential replication origin region. CDSs are boxed. Arrows below the sequences indicate the 16 to18-bp AT-rich direct repeats. Inverted repeats of 15 and 21 bases are indicated by dashed lines.

Immediately downstream of CDS 001, there is an AT-rich region with five direct repeats and a set of inverted repeats (Fig. 3). The former is possibly an iteron, a region with AT-rich direct repeats that serves as the binding site for the plasmid-encoded Rep initiator protein (55). Such a replication origin containing an iteron is common to a large group of plasmids including the IncQ group plasmids (RSF1010, R1162, and R300B), P1, F, pSC101, R6K, Rts1, pColIV-K30, RK2, RP4, pCU1, pSa, and pPS10, as well as in the lambda phage genome and in bacterial chromosomes (31, 55). Among these, an IncP group plasmid, RK2, has the iteron-type replication origin downstream of its Rep gene (28, 43). The iteron-like structure of the AT-rich region in pAQU1 and its position immediately downstream of the putative Rep protein gene suggests this region may correspond to the replication origin of pAQU1.

The plasmid partitioning system consists of two proteins encoded by independent genes often known as *parA* and *parB* (*korB*), and a cis-acting centromere-like site, *parS*(14). The deduced amino acid sequences of CDSs 037 and 038 located 30-kb downstream of CDS 001 were similar to ParA and ParB (KorB). The identities of the deduced amino acid sequences of CDSs 037 and 038 to those of ParA and ParB (KorB) proteins of the IncA/C plasmids were 75–76% and 54–58%, respectively, compared with those derived from *P. damselae* subsp. *piscicida*, (AB277723, AB277724), *Yersinia ruckeri* (CP000602), *Yersinia pestis* biovar Orientalis (CP000603), *Salmonella enterica* subsp. enterica serovar Newport (CP000604), *E. coli* (FJ621586, FJ621588, FJ705806), *Salmonella enterica* (AB277724), *Aeromonas hydrophila* (FJ705807), *Xenorhabdus nematophila* (FN667743), and *A. salmonicida* (CP000645).

The amino acid sequence of the product of CDS 191 had 60% identity to the DNA replication terminus site-binding protein (Tus) encoded by plasmids derived from *Moritella* sp. CDS 191 had 44–48% identity at the amino acid level to Tus encoded by the group of IncA/C plasmids mentioned above (except for *A. salmonicida*). A similar degree of identity was also found between the product of pAQU1 CDS 191 and Tus encoded by an IncT plasmid derived from *Proteus vulgaris* (AP004237).

### Genes involved in conjugative transfer

The deduced amino acid sequences of 18 CDSs (062, 063, 067 through 073, 075, 076, 078, 079, 081, 082, and 202 through 204) showed 50–82% identity to those of the apparatus of the type IV secretion system (Table 1). In addition, there were two CDSs (065 and 091) whose deduced amino acid sequences were similar to those of the proteins encoded by genes *s043* and *s063* found in SXT. SXT/R391 is an integrative conjugative element (ICE) of less than 100 kbp that has been detected in clinical and environmental isolates (4). *s043* and *s063* have been shown to be essential for conjugative transfer of SXT (58). pAQU1 has all 20 CDSs encoding the proteins that appear to be necessary for the type IV secretion system-dependent transfer, suggesting that conjugative transfer of this plasmid is mediated by this system. Furthermore, observation of the transfer of pAQU1 from transconjugants to the alternative recipient *E. coli* W3110Rif^r^ (data was not shown) suggested that sufficient genes for this system were encoded on the plasmid. IncA/C plasmids also have both genes of the type IV secretion system proteins and genes similar to *s043* and *s063*. Thus, IncA/C plasmids and SXT/R391 may have evolved from a common ancestral genetic element (58). Because pAQU1 shares the organization of the 20 CDSs encoding proteins involved in conjugative transfer of the IncA/C plasmids and SXT/R391, pAQU1 may have evolved from the same ancestor.

**Fig. 4:**
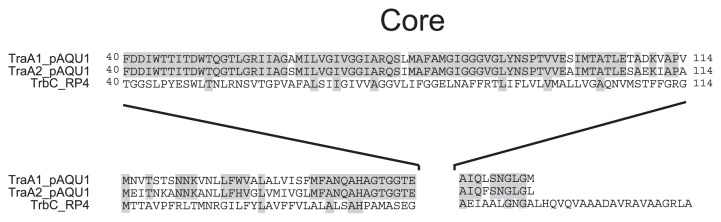
Alignment of TraA homologues. Conserved amino acids are indicated by gray boxes.

Interestingly, pAQU1 has a pair of *traA* genes located together, *traA1* and *traA2*, corresponding to CDSs 072 and 073, respectively. The TraA protein, a TrbC/VirB2 homologue, is a subunit of bacterial pili serving as a pathway for DNA transfer from a donor cell to a recipient cell (27). The deduced amino acid sequences of TraA1 and TraA2 proteins encoded by pAQU1 are identical in length and 81% similar (101 residues/124). To incorporate TraA into mature pili, it is processed by removal of the 36-residue signal peptide and the 27-residue carboxyl terminal polypeptide (22). Amino acid sequences of the processed TraA1 and TraA2 are highly conserved with only two amino acid differences (Fig. 4), suggesting they can both serve as functional subunits of pili. Phylogenetic analysis showed that pAQU1 TraA1 and TraA2 belong to an independent cluster separated from other known TraA proteins (data not shown). It may be possible *traA1* and *traA2* were generated by gene duplication within the plasmid. Another example of *traA* gene duplication is shown in the plasmid of *Xenorhabdus nematophila* ATCC 19061 (accession no. FN667743). Although the biological significance of *traA* gene duplication is unknown, it is possible that this may lead to an increased level of TraA expression and pili formation.

Plasmids encoding the proteins of the type IV secretion system have previously been found in pathogens for fish cultured in either seawater or freshwater, *e.g. Aeromonas hydrophila*, *P. damselae* subsp. *piscicida*, *Y. ruckeri* and *A. salmonicida*(13, 24, 45, 57). Our data show for the first time that bacteria carrying a conjugative plasmid could directly be isolated from seawater. Thus, conjugative transfer of the plasmid that carries multiple drug resistance genes may take place in the aquaculture environment and play an important role in the generation of multi-drug resistant pathogens.

### Drug resistance genes

Deduced amino acid sequences of nine CDSs (084, 161, 165, 166, 167, 177, 179, 180 and 181) were found to be homologous to those of known drug resistance genes.

The nucleotide sequence of CDS 084 was identical to known but officially unnamed beta-lactamase genes (AB083415, AB453229) (Table 1) found in the plasmids of fish pathogen *P. damselae* subsp. *piscicida* strains isolated from cultured yellowtail (33). CDS 084 encodes a 224 aminoacid protein that has the serine-threonine-phenylalanine-lysine (STFK) tetrad active site characteristic of serine beta-lactamases (21). Further, its 55% similarity to a carbenicillin-hydrolyzing class A beta-lactamase (CARB-9) (44) and the presence of the arginine-serine-glycine (RSG) motif instead of the K-T/S-G motif of other class A beta-lactamases suggests that the product of CDS 084 is a novel carbenicillinase gene (*bla*_CARB-9_-like) belonging to the CARB family (44). As described above, a previous work detected this gene in some strains of *P. damselae* subsp. *piscicida*, suggesting that these carbenicillinase genes may be widely distributed among *Photobacterium* in the marine environment.

CDS 161 encodes a 405 amino-acid protein with 99% identity to FloR. FloR is a drug transporter of the major facilitator superfamily known as chloramphenicol and florfenicol resistance proteins. It is found in many Gram-negative bacteria including *E. coli*, *K. pneumoniae*, *S. enterica*, and *V. cholerae*. It is not uncommon for the transposase genes to be found upstream or downstream of the *floR* gene (51). DNA sequences upstream and downstream of CDS 161 are highly similar to the *floR* genes found in other plasmids and in the SXT element (51), appearing to contain putative transposase genes. CDS 161 is located in a region with a GC% much higher than the average of the entire plasmid, suggesting that the CDS may have been exogenously introduced to pAQU1 by transposition.

Based on the deduced amino acid sequence, CDSs 165 and 166 showed 63% and 51% similarity to macrolide 2′-phosphotransferase Mph(A) and macrolide efflux pump Mef(A), respectively (9, 47), where the latter belongs to the major facilitator superfamily of efflux pumps. There is a standardizing rule for the nomenclature of macrolide resistance genes that the new gene must have <80% identity at the amino acid level with known macrolide resistance genes (47, 48); therefore, CDSs 165 and 166 are possibly new macrolide resistance genes, though their function needs to be confirmed to name them as new genes. CDS 167 encodes dihydropteroate synthase *sul2* with 99% identity. This gene is found in many IncA/C plasmids (24, 57) and encodes dihydropteroate synthase with low affinity to sulfonamide, conferring resistance to the drug (53). The deduced amino acid sequences of CDSs 177 and 181 showed 100% similarity to the ribosomal protection protein and the efflux protein encoded by *tet*(M) and *tet*(B), respectively (48). The BLAST search identified the deduced amino acid sequences of CDSs 179 and 180 as *tetD* and *tetC*(5), respectively, however, these were not characterized as tetracycline resistance determinants (18, 42), so we did not classify them as antibiotic resistance genes. Thus, many of the antibiotic resistance genes encoded on pAQU1 have quite high similarity to other corresponding genes, while some appear to be new genes.

Consistent with the annotation of the putative drug resistance genes, increases in MICs of the transconjugant were observed for ampicillin, carbenicillin, erythromycin, florfenicol and chloramphenicol, and plasmid-mediated transfer of drug resistance was confirmed (Table 2). Due to the inherent insusceptibility of the recipient strain of *E. coli* W3110, the transmission of resistance to sulfamethoxazole and sulfamonomethoxine could not be tested. Interestingly, all of the drug resistance genes found in pAQU1 had a corresponding drug that has been permitted for use in aquaculture in Japan (32). Oxytetracycline and flumequine had indeed been administered in the aquaculture area where *P. damselae* subsp. *damselae* strain 04Ya311 was obtained (38). Most of the drug resistance genes are localized in between 140,000 and 160,000 positions and are flanked by transposase genes, apparently making an island region. The sequential transposition events and drug selection in the environment possibly played a role in constructing a multi-drug resistance island in the plasmid. Thus, plasmids possessed by bacteria in the aquaculture environment serve as an important reservoir for the various antibiotic resistance genes. The transfer of resistance genes among plasmids and/or chromosomes of different bacteria may also occur in the environment. Impact of this ability to transfer among bacteria needs to be investigated in relation to the emergence of resistance in fish pathogens (23) or aquatic environmental bacteria (15, 38).

### Phylogenetic position of pAQU1

To characterize and classify the plasmids derived from human clinical isolates, replicon typing using DNA hybridization or PCR can be used (10). However, replicon typing tends to be difficult to apply to plasmids isolated from marine bacteria because their classification does not fit the plasmids derived from human clinical isolates (11, 54). Recently, comparison of the primary structures of *traI* gene products (relaxase) was successfully used for phylogenetic analysis and classification of the conjugative transfer system in six MOB families. Therefore, comparison of relaxases may be useful for plasmid classification (16). Relaxase is an essential enzyme for the type IV secretion system and cleaves at a specific site called *oriT* when it is transferred to another bacterial cell by conjugation. Because the replicon type for pAQU1 could not be determined as mentioned above, phylogenetic analysis of pAQU1 was performed based on the deduced amino acid sequence of relaxase it encodes.

**Fig. 5:**
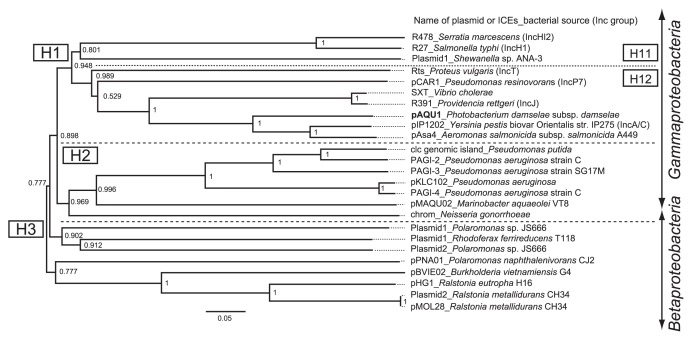
Phylogenetic tree of relaxases (TraI) from plasmids and SXT/R391 that belong to the MOB_H_ family.

**Fig. 6:**
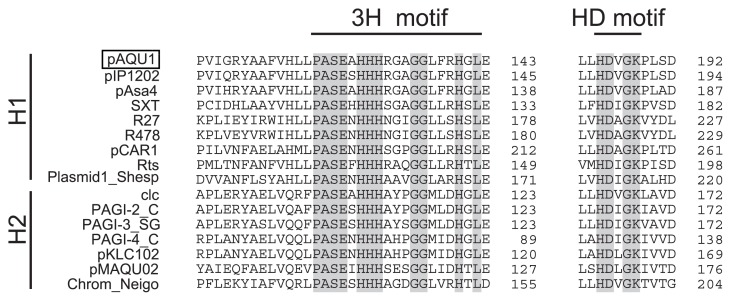
Conserved sequence motifs in relaxases (TraI) grouped in the MOB_H_ family. Both of the 3H and HD hydrolase motifs indicated by gray boxes are conserved in the relaxase encoded by pAQU1.

The data indicate that pAQU1 is a member of the MOB_H_ family and grouped in MOB_H12_ together with IncJ, IncT, IncP7, and IncA/C (Fig. 5). The relaxase encoded by pAQU1 has both 3H and HD motifs [(H/Q)-X_2_-PASE-X-HHH-X_3_-GG-X_3_-H-X-L and (L/V)-X-HD-(A/V/L/I)-GK, respectively] known to be shared by relaxases of the MOB_H1_ and MOB_H2_ clades (Fig. 6). Among the MOB_H12_ plasmids, pAQU1 is most closely related to IncA/C plasmid and SXT/R391. In addition to the organizations of the 20 CDSs encoding the type IV secretion system proteins, the primary structure of the relaxase also demonstrated a close evolutionary relationship of pAQU1 with the Inc A/C plasmid and SXT/R391; however, the DNA sequence of the *traI* gene of pAQU1 is clearly different from those of the IncA/C plasmids and SXT/R391. Recently, evidence suggested that the IncA/C plasmids (7, 25, 30, 57) and SXT/R391 (4) found in various ecological niches may be responsible for the generation of multi-drug resistant bacteria. Similarly, pAQU1, also classified in MOB_H12_, may play a similar role in the aquaculture environment, as well as in clinical settings. To examine this possibility, further investigations concerning the distribution of the ‘pAQU1-type’ plasmid will be necessary.

## Supplementary Material



## Figures and Tables

**Table 1:** Classification of CDSs identified in pAQU1

Putative function	Similarity observed	CDS no. and gene name	Identity (%)(number of identical amino acids/total length)	Query coverage (%)	Protein similarity observed
Replication, partition and termination	Yes	001 (*repA*)	34 (76/226)	62	Putative replication protein
		037 (*parA*)	76 (177/232)	91	Partition protein ParA
		038 (*parB*)	57 (233/407)	100	Transcriptional repressor protein KorB
		191 (*ter*)	60 (171/286)	95	DNA replication terminus site-binding protein

DNA processing	Yes	033	96 (1054/1101)	99	Type I restriction enzyme
		058	80 (579/725)	98	DNA topoisomerase III
		108	76 (224/296)	100	DNA modification methylase
		113	41 (55/134)	97	Type IIA topoisomerase
		128	79 (158/201)	72	5′-nucleotidase
		134	60 (353/592)	98	DNA primase
		156	42 (81/193)	94	DNA topoisomerase I
		176	90 (388/430)	100	RNA-directed DNA polymerase
		201	71 (342/485)	96	DNA helicase
		229	74 (306/412)	99	DNA methyltransferase

Conjugative transfer	Yes	062 (*traI*)	50 (528/1058)	99	TraI
		063 (*traD*)	81 (504/621)	99	TraD
		065 (*s043*)	60 (116/194)	100	Hypothetical protein
		067 (*traL*)	75 (157/209)	100	TraL
		068 (*traE*)	68 (141/208)	99	TraE
		069 (*traK*)	61 (192/314)	99	TraK
		070 (*traB*)	57 (251/443)	98	TraB
		071 (*traV*)	63 (120/189)	98	TraV
		072 (*traA1*)	70 (89/127)	100	TraA
		073 (*traA2*)	67 (82/123)	100	TraA
		075 (*dsbC*)	60 (122/203)	85	DsbC
		076 (*traC*)	77 (629/814)	99	TraC
		078 (*trhF*)	62 (106/170)	99	TrhF
		079 (*traW*)	58 (242/417)	98	TraW
		081 (*traU*)	82 (276/335)	100	TraU
		082 (*traN*)	66 (613/929)	99	TraN
		091 (*s063*)	76 (156/205)	91	Hypothetical protein
		202 (*traF*)	55 (174/318)	88	TraF
		203 (*traH*)	70 (337/479)	99	TraH
		204 (*traG*)	71 (860/1214)	99	TraG

Transposition	Yes	050	98 (365/373)	100	ISVha1 (ISAs1 family)
		056	95 (229/240)	100	ISVba2 (IS5 family, IS903 group)
		157	100 (312/312)	100	ISVsa5 (IS4 family, IS10 group)
		158	100 (311/311)	89	ISShfr9 (Tn3 family)
		159	99 (238/239)	100	ISVsa3 (IS91 family)
		163	100 (497/497)	100	ISVsa3 (IS91 family)
		172	98 (161/165)	97	ISVsa5 (IS4 family, IS10 group)
		173	99 (236/237)	100	ISVsa5 (IS4 family, IS10 group)
		174	99 (586/587)	97	ISShfr9 (Tn3 family)
		178	99 (401/402)	100	ISVsa5 (IS4 family, IS10 group)
		187	96 (298/312)	100	ISVsa5 (IS4 family, IS10 group)
		189	99 (233/234)	93	ISVsa5 (IS4 family, IS10 group)

Integration	Yes	024	98 (309/315)	100	Integrase core domain
		025	94 (186/197)	100	Integrase family protein
		089	78 (420/537)	98	Integrase family protein
		150	60 (191/321)	99	Integrase family protein

Antibiotic resistance	Yes	084 (*bla*_CARB-9_-like)	100 (223/223)	100	Beta lactamase class A (carbenicillinase)
		161 (*floR*)	99 (402/404)	100	Florfenicol/chloramphenicol resistance protein
		165 (*mph*(A)-like)	63 (186/293)	99	Macrolide 2′-phosphotransferase Mph(A)
		166 (*mef*(A)-like)	51 (208/407)	99	Macrolide efflux pump Mef(A)
		167 (*sul2*)	99 (270/271)	100	Dihydropteroate synthase
		177 (*tet*(M))	100 (639/639)	100	Ribosomal protection protein TetM
		181 (*tet*(B))	100 (401/401)	100	Tetracycline efflux protein TetB

Others	Yes	002	45 (640/1419)	99	Rhs family protein
		005	52 (154/296)	97	Rhs family protein
		019	98 (235/240)	92	LabA-like protein
		029	95 (483/508)	100	Type I restriction enzyme M protein
		031	53 (225/428)	99	ATP-like protein
		034	98 (252/258)	100	Metal-dependent hydrolase
		080	40 (86/217)	92	Cyclic diguanylate phosphodiesterase domain-containing protein
		096	78 (249/320)	99	Aerobic cobaltochelatase subunit Cobs
		102	66 (400/605)	100	Von Willebrand factor type A domain-containing protein
		116	58 (43/74)	97	Putative redox protein, regulator of disulfide bond formation
		131	47 (131/278)	92	NgrC
		162	100 (101/101)	100	LysR family transcriptional regulator
		170	100 (237/237)	100	Mobilization protein B
		175	98 (49/50)	80	Tn*916*, transcriptional regulator (ORF9)
		182	100 (208/208)	100	Tetracycline repressor protein TetR
		183	100 (207/207)	100	ArsR family transcriptional regulator
		186	99 (346/347)	96	Sodium/glutamate symport carrier protein
		188	100 (58/58)	100	Sodium/glutamate symporter (fragment)
		206	68 (48/71)	86	Ner-like DNA-binding protein
		209	72 (127/177)	92	Putative regulator protein
		222	72 (228/316)	98	Periplasmic serine protease
		223	57 (165/292)	100	DSBA-like thioredoxin domain protein

Unknown	Yes or No^a^	003, 004, 006–018, 020–023, 026–028, 030, 032, 035, 036, 039–049, 051–055, 059, 060, 061, 064, 066, 074, 077, 083, 085–088, 090, 091–095, 097–101, 103–107, 109–112, 114, 115, 117–127, 129, 130, 132, 133, 135–149, 151–155, 160, 164, 168, 169, 171, 179, 180, 184, 185, 190, 192–200, 205, 207, 208, 210–221, 224–228, 230–235	ND^b^	ND	ND

a Yes, similarity was observed with hypothetical proteins; No, similarity was not observed with known or hypothetical proteins.

b ND, not determined.

**Table 2:** Susceptibility of the donor strain of *P. damselae* subsp. *damselae* 04Ya311, the transconjugant TJ311W2, and *E. coli* W3110 to antimicrobial agents

Strain name	Species	Minimal inhibitory concentration (μg/ml)^a^							

		AMP	CAR	TET	ERY	CHL	FLO	SMXZ	SMX
04Ya311	*P. damselae* subsp. *damselae*	>128	>128	8	128	8	16	>512	>512
TJ311W2	*E. coli*	64	>128	16	128	8	16	>512	>512

W3110	*E. coli*	0.125	1	<0.125	32	0.5	1	>512	>512

a AMP, ampicillin; CAR, carbenicillin; TET, tetracycline; ERY, erythromycin; CHL, chloramphenicol; FLO, florfenicol; SMXZ, sulfamethoxazole; SMX, sulfamonomethoxine

## References

[1] Alcaide E (2003). Numerical taxonomy of *Vibrionaceae* isolated from cultured amberjack (*Seriola dumerili*) and surrounding water. Curr Microbiol.

[2] Barber GR, Swygert JS (2000). Necrotizing fasciitis due to *Photobacterium damsela* in a man lashed by a stingray. N Engl J Med.

[3] Barton BM, Harding GP, Zuccarelli AJ (1995). A general method for detecting and sizing large plasmids. Anal Biochem.

[4] Burrus V, Marrero J, Waldor MK (2006). The current ICE age: biology and evolution of SXT-related integrating conjugative elements. Plasmid.

[5] Braus G, Argast M, Beck CF (1984). Identification of additional genes on transposon Tn*10: tetC* and *tetD*. J Bacteriol.

[6] Cabello FC (2006). Heavy use of prophylactic antibiotics in aquaculture: a growing problem for human and animal health and for the environment. Environ Microbiol.

[7] Call DR, Singer RS, Meng D (2010). *bla*_CMY-2_-positive IncA/C plasmids from *Escherichia coli* and *Salmonella enterica* are a distinct component of a larger lineage of plasmids. Antimicrob Agents Chemother.

[8] Capella-Gutiérrez S, Silla-Martínez JM, Gabaldón T (2009). trimAl: a tool for automated alignment trimming in large-scale phylogenetic analyses. Bioinformatics.

[9] Clancy J, Petitpas J, Dib-Hajj F, Yuan W, Cronan M, Kamath AV, Bergeron J, Retsema JA (1996). Molecular cloning and functional analysis of a novel macrolide-resistance determinant, *mef* A, from *Streptococcus pyogenes*. Mol Microbiol.

[10] Couturier M, Bex F, Bergquist PL, Maas WK (1988). Identification and classification of bacterial plasmids. Microbiol Rev.

[11] Dahlberg C, Linberg C, Torsvik VL, Hermansson M (1997). Conjugative plasmids isolated from bacteria in marine environments show various degrees of homology to each other and are not closely related to well-characterized plasmids. Appl Environ Microbiol.

[12] Fraser SL, Purcell BK, Delgado B, Baker AE, Whelen AC (1997). Rapidly fatal infection due to *Photobacterium*(*Vibrio*) *damsela*. Clin Infect Dis.

[13] Fricke WF, Welch TJ, McDermott PF, Mammel MK, LeClerc JE, White DG, Cebula TA, Ravel J (2009). Comparative genomics of the IncA/C multidrug resistance plasmid family. J Bacteriol.

[14] Funnell BE, Slavcev RA, Funnell BE, Phillips GJ (2004). Partition systems of bacterial plasmid. Plasmid Biology.

[15] Furushita M, Shiba T, Maeda T (2003). Similarity of tetracycline resistance genes isolated from fish farm bacteria to those from clinical isolates. Appl Environ Microbiol.

[16] Garcillán-Barcia MP, Francia MV, de la Cruz F (2009). The diversity of conjugative relaxases and its application in plasmid classification. FEMS Microbiol Rev.

[17] Goodell KH, Jordan MR, Graham R, Cassidy C, Nasraway SA (2004). Rapidly advancing necrotizing fasciitis caused by *Photobacterium*(*Vibrio*) *damsela*: a hyperaggressive variant. Crit Care Med.

[18] Griffith KL, Becker SM, Wolf RE (2005). Characterization of *TetD* as a transcriptional activator of a subset of genes of the *Escherichia coli* SoxS/MarA/Rob regulon. Mol Microbiol.

[19] Hayashi K, Morooka N, Yamamoto Y (2006). Highly accurate genome sequences of *Escherichia coli* K-12 strains MG1655 and W3110. Mol. Syst. Biol.

[20] Johnson TJ, Nolan LK (2009). Plasmid replicon typing. Methods Mol Biol.

[21] Joris B, Ledent P, Dideberg O, Fonze E, Lamotte-Brasseur J, Kelly JA, Ghuysen JM, Frere JM (1991). Comparison of the sequences of class A β-lactamases and of the secondary structure elements of penicillin-recognizing proteins. Antimicrob Agents Chemother.

[22] Kalkum M, Eisenbrandt R, Lurz R, Lanka E (2002). Tying rings for sex. Trends Microbiol.

[23] Kim SR, Nonaka L, Suzuki S (2004). Occurrence of tetracycline resistance genes *tet*(M) and *tet*(S) in bacteria from marine aquaculture sites. FEMS Microbiol Lett.

[24] Kim MJ, Hirono I, Kurokawa K, Maki T, Hawke J, Kondo H, Santos MD, Aoki T (2008). Complete DNA sequence and analysis of the transferable multiple-drug resistance plasmids (R Plasmids) from *Photobacterium damselae* subsp. *piscicida* isolates collected in Japan and the United States. Antimicrob Agents Chemother.

[25] Kumarasamy KK, Toleman MA, Walsh TR (2010). Emergence of a new antibiotic resistance mechanism in India, Pakistan, and the UK: a molecular, biological, and epidemiological study. Lancet Infect Dis.

[26] Kawanishi M, Kojima A, Ishihara K (2004). Quality control ranges of minimum inhibitory concentrations for *Lactococcus garvieae* and *Photobacterium damselae* subsp. *piscicida*. Fish Pathol.

[27] Lawley TD, Klimke WA, Gubbins MJ, Frost LS (2003). F factor conjugation is a true type IV secretion system. FEMS Microbiol Lett.

[28] Llanes C, Gabant P, Couturier M, Michel-Briand Y (1994). Cloning and characterization of the Inc A/C plasmid RA1 replicon. J Bacteriol.

[29] Love M, Teebken-Fisher D, Hose JE, Farmer JJ, Hickman FW, Fanning GR (1981). *Vibrio damsela*, a marine bacterium, causes skin ulcers on the damselfish *Chromis punctipinnis*. Science.

[30] McIntosh D, Cunningham M, Ji B (2008). Transferable, multiple antibiotic and mercury resistance in Atlantic Canadian isolates of *Aeromonas salmonicida* subsp. *salmonicida* is associated with carriage of an IncA/C plasmid similar to the *Salmonella enterica* plasmid pSN254. J Antimicrob Chemother.

[31] Meyer R (2009). Replication and conjugative mobilization of broad host-range IncQ plasmids. Plasmid.

[32] Ministry of Agriculture, Forestry and Fisheries (2011). Protocols for the use of antimicrobial agents in aquaculture.

[33] Morii H, Bharadwaj MS, Eto N (2004). Cloning and nucleotide sequence analysis of the ampicililin resistance gene on a conjugative R plasmid from the fish pathogen *Photobacterium damselae* subsp. *piscicida*. J. Aquat. Anim Health.

[34] Nakamura Y, Uchihira M, Ichimiya M, Morita K, Muto M (2008). Necrotizing fasciitis of the leg due to *Photobacterium damsela*. J Dermatol.

[35] National Committee for Clinical Laboratory Standards (NCCLS) (2003). Methods for Dilution Antimicrobial Susceptibility Tests for Bacteria That Grow Aerobically Approved standard M7-A6.

[36] Neela FA, Nonaka L, Rahman MH, Suzuki S (2009). Transfer of the chromosomally encoded tetracycline resistance gene *tet*(M) from marine bacteria to *Escherichia coli* and *Enterococcus faecalis*. World J Microbiol Biotechnol.

[37] Noguchi H, Taniguchi T, Itoh T (2008). MetaGeneAnnotator: detecting species-specific patterns of ribosomal binding site for precise gene prediction in anonymous prokaryotic and phage genomes. DNA Res.

[38] Nonaka L, Ikeno K, Suzuki S (2007). Distribution of tetracycline resistance gene, *tet*(M), in Gram-positive and Gram-negative bacteria isolated from sediment and seawater at a coastal aquaculture site in Japan. Microbes Environ.

[39] Osorio CR, Toranzo AE, Romalde JL, Barja JL (2000). Multiplex PCR assay for *ureC* and 16S rRNA genes clearly discriminates between both subspecies of *Photobacterium damselae*. Dis Aquat Organ.

[40] Pedersen K, Dalsgaard I, Larsen JL (1997). *Vibrio damsela* associated with diseased fish in Denmark. Appl Environ Microbiol.

[41] Pedersen K, Skall HF, Lassen-Nielsen AM, Bjerrum L, Olesen NJ (2009). *Photobacterium damselae* subsp. *damselae*, an emerging pathogen in Danish rainbow trout, *Oncorhynchus mykiss*(Walbaum), mariculture. J Fish Dis.

[42] Pepe CM, Suzuki C, Laurie C, Simons RW (1997). Regulation of the “tetCD” genes of transposon Tn*10*. J Mol Biol.

[43] Perri S, Helinski DR, Toukdarian A (1991). Interactions of plasmid-encoded replication initiation proteins with the origin of DNA replication in the broad host range plasmid RK2. J Biol Chem.

[44] Petroni A, Melano RG, Saka HA (2004). CARB-9, a carbenicillinase encoded in the VCR region of *Vibrio cholerae* non-O1, non-O139 belongs to a family of cassette-encoded β-lactamases. Antimicrob Agents Chemother.

[45] Reith ME, Singh RK, Curtis B (2008). The genome of *Aeromonas salmonicida* subsp. *salmonicida* A449: insights into the evolution of a fish pathogen. BMC Genomics.

[46] Richards GP, Watson MA, Crane EJ, Burt IG, Bushek D (2008). *Shewanella* and *Photobacterium* spp. in oysters and seawater from the Delaware Bay. Appl Environ Microbiol.

[47] Roberts MC, Sutcliffe J, Courvalin P, Jensen LB, Rood J, Seppala H (1999). Nomenclature for macrolide and macrolide-lincosamide-streptogramin B resistance determinants. Antimicrob Agents Chemother.

[48] Roberts MC (2005). Update on acquired tetracycline resistance genes. FEMS Microbiol Lett.

[49] Roberts MC (2008). Update on macroride-lincosamid-streptogramin, ketlide, and oxazolidinone resistance genes. FEMS microbial. Lett.

[50] Sambrook J, Russell DW (2001). Molecular Cloning: A Laboratory Manual.

[51] Schwarz S, Kehrenberg C, Doublet B, Cloeckaert A (2004). Molecular basis of bacterial resistance to chloramphenicol and florfenicol. FEMS Microbiol Rev.

[52] Shin JH, Shin MG, Suh SP, Ryang DW, Rew JS, Nolte FS (1996). Primary *Vibrio damsela* septicemia. Clin Infect Dis.

[53] Skold O (2000). Sulfonamide resistance: mechanisms and trends. Drug Resist Updat.

[54] Sobecky PA, Mincer TJ, Chang MC, Helinski DR (1997). Plasmids isolated from marine sediment microbial communities contain replication and incompatibility regions unrelated to those of known plasmid groups. Appl Environ Microbiol.

[55] Solar GD, Giraldo R, Ruiz-Echevarría MJ, Espinosa M, Díaz-Orejas R (1998). Replication and control of circular bacterial plasmids. Microbiol Mol Biol Rev.

[56] Tamura K, Peterson D, Peterson N, Stecher G, Nei M, Kumar S (2011). MEGA5: Molecular evolutionary genetics analysis using maximum likelihood, evolutionary distance, and maximum parsimony methods. Mol Biol Evol.

[57] Welch TJ, Fricke WF, McDermott PF (2007). Multiple antimicrobial resistance in plague: an emerging public health risk. PLoS One.

[58] Wozniak RA, Fouts DE, Spagnoletti M, Colombo MM, Ceccarelli D, Garriss G, Dery C, Burrus V, Waldor MK (2009). Comparative ICE genomics: insights into the evolution of the SXT/R391 family of ICEs. PLoS Genet.

[59] Yamane K, Asato J, Kawade N, Takahashi H, Kimura B, Arakawa Y (2004). Two cases of fatal necrotizing fasciitis caused by *Photobacterium damsela* in Japan. J Clin Microbiol.

[60] Zakour NB, Gautier M, Andonov R, Lavenier D, Cochet MF, Veber P, Sorokin A, Le Loir Y (2004). GenoFrag: software to design primers optimized for whole genome scanning by long-range PCR amplification. Nucleic Acids Res.

