# Supplementary material for: Novel Conjugative Transferable Multiple Drug Resistance Plasmid pAQU1 from Photobacterium damselae subsp. damselae Isolated from Marine Aquaculture Environment
Source: Microbes Environ. 2012 Mar 23;27(3):263–72. doi: 10.1264/jsme2.ME11338 (PMC4036041; doi:10.1264/jsme2.ME11338)
Supplement: Supplementary file 1 [file 27_263_s1.pdf]

Figure legends for a supplemental figure

Figure S1. Alignment of amino acid sequences from the 25 relaxases (TraI) in plasmids and SXT/R39 that belong to the MOB_H family. The amino acids sequences of the highly conserved regions (boxed) were aligned.

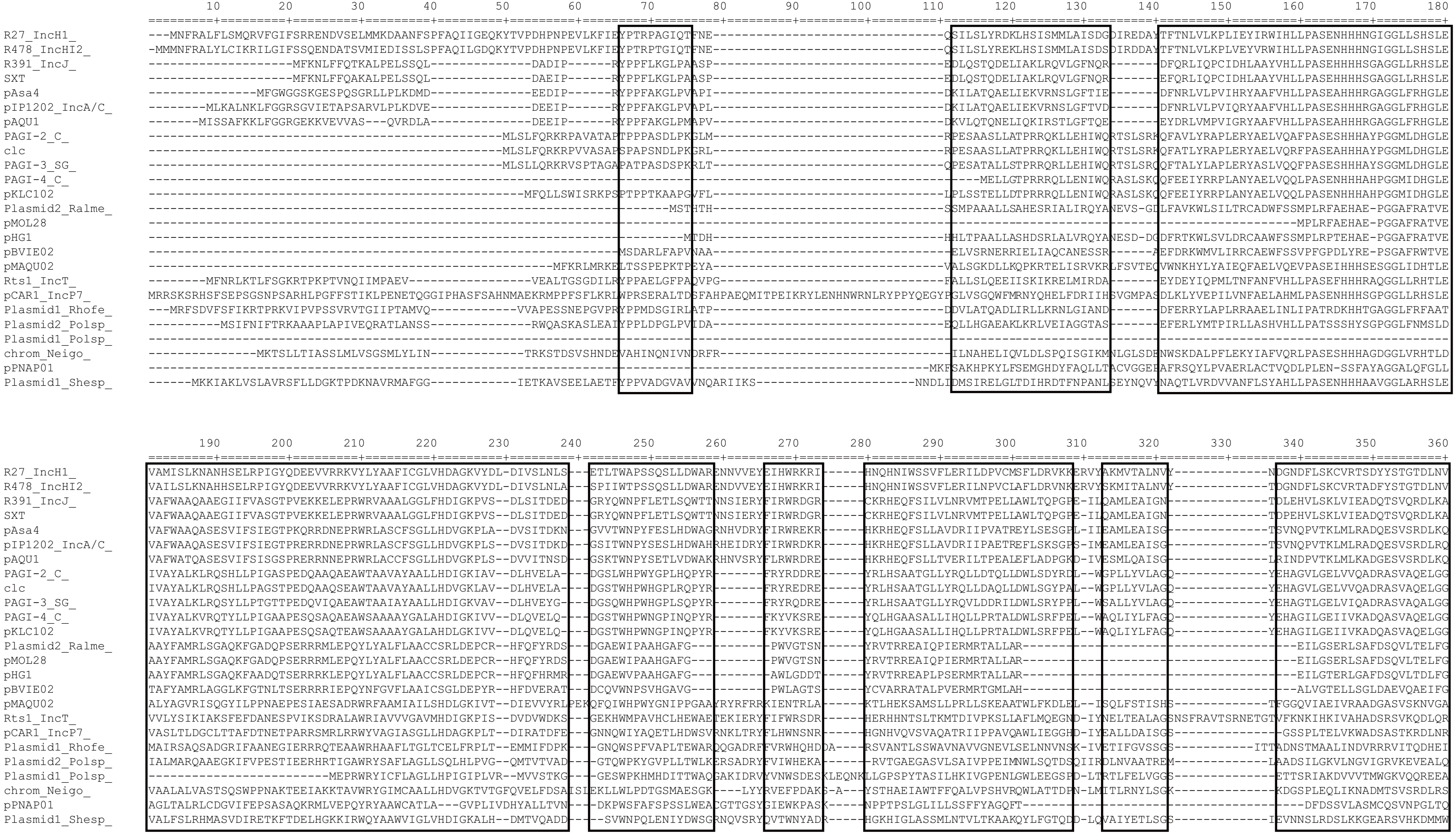

Fig. S1 Nonaka *et al.*

```
                          370       380       390       400       410       420       430       440       450       460       470       480       490       500       510       520       530       540
                  =========+=========+=========+=========+=========+=========+=========+=========+=========+=========+=========+=========+=========+=========+=========+=========+=========+=========+
R27_IncH1_        LR-DPIMGLRSNDAAARAIGTIKHNFTSININNYKTKPMHIIIVNGE---VYLNENAFLDFVLSDFAAHKFNFPQGDAG-KTVLVESLVQRGYVEPYDDERVVHYFIP-GTYSENEIASIFRNGIGKLEFYNLLKLRWIGLIFDSYKIPDSVPGLFSVNANKDFIYIDEQKTVTEYRRP
R478_IncHI2_      LR-DPIMGLRSNDAAARAISTIKHNFTSININNYNAKPMHIIIVNGE---VYLNENAFLDFVLNDFELHKYNFPQGEAG-KTVLVESLVQRGYVEPYDDERVVHYFIP-GIYSENEISNIFRNGIGKLEFYNLLKLRWIGLIFDSYKIPDSVPGLFSVNANKDFIYIDEQKTVTEYRRP
R391_IncJ_        QR-ISVDDNALGVPVERYLLDAMRRLLASSQWLVNQRDARVWLRKSNQSTHLYLVWKSAAKDIIELLAKDKIPGIPRDP---DTLADILIERGLATKSASNE--------RYESLAPEVLIK---DDKPIWLPMLHISEADLLFSSNVPSSVRLFSKSEWEATRQTQAEPQSRSSEHS--
SXT               QR-ISVDDNALGVPVERYLLDAMRRLLASSQWLVNQRDVRVWLRKSNQSTNLYLVWKSGAKEIIELLAKDKIPGIPRDP---DTLADILIERGLAIKSASNE--------RYESLAPEVLIK---DDKPIWLPMLHIVEADLLFSSNVPSGVILFNKSEWEATQQTQAEPQSRSSEHS--
pAsa4             NR-LNVDEFSYGVPVERYVFDAIRRLVKTGKWKVNEPGAKVWHLQQG---VFIAWRN-LGDLYDLISHDKIPGIPRDP---DTLADILIERGFAVPNTVQEKGDRAYY-RYWEVLPEMIQ---EAAGSIKILMLRLESNELVFTTEPPAAVAGEVVGDVADAVIELIDPDEASDADD--
pIP1202_IncA/C_   SR-LDVDEFSYGVPVERYVFDAIRRLVKTGKWKVNEPGAKVWHLNQG---VFIAWKQ-LGDLYDLISHDKIPGIPRDP---DTLADILIERGFAVPNTVQEKGERAYY-RYWEVLPEMLQ---EAAGSVKILMLRLESNDLVFTTEPPAAVAAEVVGDVEDAEIEFVDPEEADDGDDQE
pAQU1             NR-LDVDEFAYGVPVERYVFDALRRLVKTGKWKVNEPGAKVWHLHQG---VFITWKQGASDIYDLLTEDKIPGIPRDP---DTLADILIERGFALKNVLPEHGKDVFY-RYWEVIPELPQEGEEVTGKIKMQMLRLESSELVFTTEPPAAIAGEVIGDEDEQELDASELDDDAPEDG--
PAGI-2_C_         DP-ARAMAAPKHALQRKLLDGLRYLLKEELKLNQPE-ASDGWLTEDA---LWLVSKTVSDKLRAHLLSQGIDGIPANN---TAVFNVLQDHGMLQPTLDGKAVWRATV-TSNAGWTHSFTLLRLAPALIWE-AGERPAPFAGTVAIDTSPVEKHAA---------AASSPEVAAKPTP-
clc               DP-ARAMAAPKHALQRKLLDGLRYLLKEELKLNQPE-ASDGWLTEDA---LWLVSKTVSDKLRAHLLSQGIDGIPANN---TAVFNVLQDHGMLHPTTDGKAVWRATV-TSATGWSHSFTLLRLAPALIWE-PGERPVPFAGTVAIDAVPNDK-----------SVAQPAAVAETTQ-
PAGI-3_SG_        DP-ARAMAAPKHALQRKLLEGLRYLLKEELKLNQSG-ASDGWLTQDA---LWLVSKTVSDKLRAHLLAQGIDGIPANN---TAVFNVLQDHGMLQPTPDGKAIWRATV-TSTAGWAHTFTLLRLAPALIWE-GENRPEPFTGTVLIEME------------------SNDAPAEPSS-
PAGI-4_C_         NP-DRALAAPKQSLQRQLADGLRFLVKDKFKLNQPGGPSDGWLTQDA---LWLVSKPAADQLRAYLLAQGIEGVPSSN---APFFNMLQDQAVIQTNAEDKAIWTATI-DNGAGWRNKFTLLKIAPALIWADPAERPDSYSGSLVVEEGNASPEKPETTCEIPNDPIEQRQAPEAKMT-
pKLC102           NP-DRALAAPKQSLQRQLADGLRFLVKDKFKLNQPGGPSDGWLTQDA---LWLVSKPAADQLRAYLLAQGIEGVPSSN---ASFFNMLQDQAVIQTNAEDKAIWTATI-DNGAGWRNKFTLLKIAPALIWADPAERPDSYSGSLVIEEGNASSEKPETTCEIPNDSIEQRQAPEAKMT-
Plasmid2_Ralme_   ----------------------------------------------------AINPDPRPTG-LETLLHKVVRQSIDT---LTQFEIKARKAEFPPDSTPVP------------------------------------------------------------------------TAERL-
pMOL28            ----------------------------------------------------AINPDPRPTG-LETLLHKVVRQSIDT---LTQFEIKARKAEFPPDSTPVP------------------------------------------------------------------------TAERL-
pHG1              ----------------------------------------------------AINPDPRPAG-LETLLHKVVRQAIET---VTQFELKARRAAFAPDTTPAPSA-------------------------------------------------------------------QALAVDAI-
pBVIE02           ----------------------------------------------------AINPTQSPTGGVESLTHKVVRQAVAT---AAEFDRKAQRAVFEPVTFAVP------------------------------------------------------------------SAIHVAAELEP-
pMAQU02           NT-GKKADHTNTIPLYEKLIVSLRKLVNDGDLKRNKPGAAVWVTDQD---TWVVSKATMEAVRIQLINEGHSGIPKNV---VTLFGILNDHDCLVPNPDGDSVWYAEINDHGKNWQQKLTFLRFKNEIIWPTSQPDMFDGTVTPIDRNG------------------NPLEISQVVEP-
Rts1_IncT_        YSGDAVRASQTGVPVIARIVDAMRLLIKKEEWKPNQAGSPLWVTTEG---VFIVWGTAVTPIISIVKESGINVPHSAD---SLADIMLNYGLCVLNSNDSVYWRLAPHILNDKISRQNKEPKNALSCIKLVDPVVLFTDDVMPNPTSCRIKLADGWKEFTGAGLTKQKSQPSRPYVG-
pCAR1_IncP7_      GS-NNGGGNATGVPVPRLVSDAMLRLLSDGTWKINTPGGRVWVATDG---IYIVWNQGAEEIVSMLVGDGVVAIPRSP---DTLIGSLVEHGIAERAPNGDLYWLVTPHLLRKNGKGPALRCMKLVNPDILFPTTPIPPPVSIGLGKEGKQKDVIAPNDTAGAAANALSEHQEDLFG--
Plasmid1_Rhofe_   AP-ATYGKLTSGSQLEPYFMDAMRTLLRTGVWQVNTKSGRCHFGADG---FYVAWRMGSQEILGHLQTEKIAGVPTAK---ETLAEMMGRAGIISIAPDGSWIHLVRS-SVGASAPMPAVRIQSPLAILGHLEVKPVADTLRVNSAKP------------------APSVAIPLTKP-
Plasmid2_Polsp_   HP-ARFGRLTIGTQFEIHLLNALRELIEEREWKLNDPNGCLFWGSDG---LYVAWPRGLEDVTSIFAKRQLKGMPLSS---VTLAEMLGQSGVVIGKDTGVWVHDIVV---------------GAEKYVVSAMRFKDPAVLLG---------------------HLDFQPVPSP-
Plasmid1_Polsp_   RRPQAYGRLTVGTHLTPYLVGSMRSLVNDGKWKPNDGPLIVDSTG-----VYLIWPDAGEEIVRQGAREGRDGWPSSA---ATLAELLKQDGVFETSYGNDMGMTEVVDKEGNVLQSYKLKKPTTVIETYEASDYQRAAPKTLSGVLDR-----------------DPLAKAEAKAV-
chrom_Neigo_      GSRQRFSTAKRKPFIETIMETLKEMLSDRGVHFSIATTAGGDLFRKGD--RIYLMSKNVPDYIRQYLRKNQHPAAGSFPADNQRIFDTLFEYRAVIPPENDPHRAINHIEVEFTRMDGEKVRNIFSVLCFNAKTLYPDGDYPTEFLGNLSEVSAKKEIPDVVVSEDKVSTQIQGAEDNP-
pPNAP01           SS-------------------------------------------------------GESSLSKLVRVAQEKVKATEKLR-----ISRVFVPGEAQVNTVASVMQA---------------------------------------------------------------------------
Plasmid1_Shesp_   QW----DRLTGARKMPLEAAFIKRLQMMRRGWKVNEEKADIWCLGAED---VYVSYPASFNKVMSSLKEDGFNVPMQVG----KMLEMLVERQLFEPLFEDYTSGHLHPVTNPEWTAGPIKVVKPKWAGIVYGEDIPQRGIHGKISLTRDDSLLIHYADTGLITLTDTREPDPQTPANN-

                          550       560       570       580       590       600       610       620       630       640       650       660       670       680       690       700       710       720
                  =========+=========+=========+=========+=========+=========+=========+=========+=========+=========+=========+=========+=========+=========+=========+=========+=========+=========+
R27_IncH1_        VPGRESVTRVTDTVNDAIENTPQYGLQLVNGPDADSN-------NIISSENTESITDSLEESGADISNEIFETQVVTAIDTAETVNADEPEQVEEHDDRSQIHLVEQLHEMLLSAPLPHHAVINIDSVPYLDLDAAIALIPGIDEAAFCNGPFFQLTYRDGSLDGMWIVRDVNNLRLIQL
R478_IncHI2_      VPGRDVITKITDTVETAVLKVNDLGRSSAS-IDVD-------IHSKKNEGSSDDFEKKAESDNEIDNDTQIVKSEGEEAADPVIPDIEESE-DESAKDTESHVLVNQLHELLLSAPLSNDYIVCVDAVPYLNIDTTMALLPGLDEKAFSEEPYFQLTFREGSLDGMWIVRDIDDLRLVQL
R391_IncJ_        --------------------------------------------DLPEASSSIEHRKSTESPSTNSSEQDDELRLASDVNNPQATEN-------------------APGDGCEKPNNSYDGAISNNVNQYDTEALN--------------------------------------------
SXT               --------------------------------------------DLPEASSSIELSNSAESPSTNSSDQDDELRLSSDVNHLQATEN-------------------APGDECEKPNNSYDGAISNNVNQHDAEALN--------------------------------------------
pAsa4             ESASGMSESLLSAEQEAMNALAGLGFGDAMEILGGTASDDNATQPAPVVDTTPPAKPAKGAGGRGGKNQAK--KTAPAAPEQQQPEP--------SPQEIAKSTPPLAVENPLQAIMDVGGGLGGLDFPFDAFVAGTSMSDEPSQALAPEPKQ------DVANPDNPD-TPNLGLMNQVF
pIP1202_IncA/C_   EGEAALNDDMLAAEQEAEKALAGLGFGDAMEMLKSTS---DAVEEKPEQKDAGPTESSKPDAGKKGKPQSKPGKAKPKSDTEKQPHKPEAK-EDLSPQDIAKNAPPLANDNPLQALKDVGGGLGDIDFPFDAFNAS---AETTSTDATNSEIP------DVAMPGKQEEQPKQDFVPQEQ
pAQU1             ----DENSDSLTATPEPIGSLD-DGF-----------------EIPDFMAAPPSTDSQSSEPKGGTSTDT--KTSKNDDKADAPKEP-------SAQEIAQTAAPLALDDPLKGLKDAGGALGVCDFPFDAFGLNTEPDTTQKDKAETPNVPTEQSEEDTSLPGIDNIRSGKKRQPKAS
PAGI-2_C_         ------------------------------------------------------------------------------------------------------------------------------------------------------------------------------------
clc               ------------------------------------------------------------------------------------------------------------------------------------------------------------------------------------
PAGI-3_SG_        ------------------------------------------------------------------------------------------------------------------------------------------------------------------------------------
PAGI-4_C_         ------------------------------------------------------------------------------------------------------------------------------------------------------------------------------------
pKLC102           ------------------------------------------------------------------------------------------------------------------------------------------------------------------------------------
Plasmid2_Ralme_   ------------------------------------------------------------------------------------------------------------------------------------------------------------------------------------
pMOL28            ------------------------------------------------------------------------------------------------------------------------------------------------------------------------------------
pHG1              ------------------------------------------------------------------------------------------------------------------------------------------------------------------------------------
pBVIE02           ------------------------------------------------------------------------------------------------------------------------------------------------------------------------------------
pMAQU02           ------------------------------------------------------------------------------------------------------------------------------------------------------------------------------------
Rts1_IncT_        --------------------------------------------EKPPERVLNVDPDILKGGSLPPTDDLPPDGTVVKRGTVIEQKNIVDHMLRMNLLSPENAAAIKSRNENEAKAKAESQDVEASPKPAETSVPAP-------------------------------------------
pCAR1_IncP7_      ----------------------------------------------ADLTGDSSGDTDADSGPTPPKESASSKPARKKKAAPRQAAPVPTTASSGVSLKQAQNPVEDAPVNVDSESAADETAVIVSSQPEEALDAE--------------------------------------------
Plasmid1_Rhofe_   ------------------------------------------------------------------------------------------------------------------------------------------------------------------------------------
Plasmid2_Polsp_   ------------------------------------------------------------------------------------------------------------------------------------------------------------------------------------
Plasmid1_Polsp_   ------------------------------------------------------------------------------------------------------------------------------------------------------------------------------------
chrom_Neigo_      -----------------------------------------VTMTKDKMDFLSIQPVTAEQPPLPEKNQTETANSAFDNSMNTAAPAENKSSESEKAKEPGYGTIDNLIENFNLIEADSEQTESTKSEAETAENAVN-------------------------------------------
pPNAP01           ------------------------------------------------------------------------------------------------------------------------------------------------------------------------------------
Plasmid1_Shesp_   --------------------------------------TAAPTQPAPAPTPTPAVAPVQQNGSQTNQSKAQTKKPKKQSARASTNTPAPAPAPATTPSEPAPEKSQVETQVEPEQSNTRAPAPDTKGIVFKNMNDTPIVSEPAEAAVEITEPIAQEMP----------------------
```

Fig. S1 (continued) Nonaka *et al.*

```
                             730       740       750       760       770       780       790       800       810       820       830       840       850       860       870       880       890       900
                    =========+=========+=========+=========+=========+=========+=========+=========+=========+=========+=========+=========+=========+=========+=========+=========+=========+=========+
R27_IncH1_          GDNCAGMQVSTSEPRNTSSLKSLFDTS--------------------------------------------------------MYQPLDIPEAPSVNEAASPPQTPLELPQRLNAPVAEEASSVAEQTNAHSE--PDSVIATEYEQYGHLLEETLDS----DGEAYSDLIASDSTEAEY
R478_IncHI2_        GDNCAGFQLTYHEPRRPTTLKSLFNTS--------------------------------------------------------MYQALVINDESSVENSAPRPKQTLELPPPRVNAVEEHSGDVEYHGTDSASATGPLKTEAVEYEHYQHLFEKEDEEHEIIDYTDFSQLSVSRPEVGSC
R391_IncJ_          ---------------------------------------------------------------------------------------------------------LPESLAWLPEASSALVMVGEQILIRYPDAVRPWCAPRKLLAELSQLDWLELDPANPTRKARTVTTNDGVQEQGLLLKVS
SXT                 ---------------------------------------------------------------------------------------------------------LPESLAWLPEASSALVMVGEQVLIRYPDAVRHWCAPRKLLAELSRLDWLELDPANPTRKARAVTTKGGVQEQGLLLKVS
pAsa4               PNFMNDESTGDVGGMDLPPAWAIEEIP------SFHAYEGEVGQPGNQFVSEELAG-----------------ADAPDAKGELLTLMATFGEAGPILERAILPVLEGKATLGEVLCLMKGQAVILYPEGARALGAPSEILTALFHAQAIVPDPVMSGRKVQDFNGVKAIVLTEQLSSAF
pIP1202_IncA/C_     NSLQGDDFP-MFGGSDEPPSWAIEPLP------MLTDAP-EQPTHTPEMPHTDNVN-----------------QHEKDAKTLLVEMLSGYGEASALLEQAIMPVLEGKTTLGEVLCLMKGQAVILYPDGARSLGAPSEVLSKLSHANAIVPDPIMPGRKVRDFSGVKAIVLAEQLSDAV
pAQU1               ANTAASLSPNEFFGPEPSAIFDAPPMPPEDMFAPIDAVPLDAYDYSHEMEEREEFVPTEHFQDAETYSFSQQDKSSDVDVRGHLENVLSGYGAAESILKEAIIPVLDGHKTLGEVLCFMKKKAVILYPEGARALGAPAEVLNTLFDAGAIVPDPVHPNRKVHDFSGIKGLLLSSDISDVI
PAGI-2_C_           --------------------------------------------------------------------------------------------------GGQESPPWEGGSAADPIIAPLAEAMPDVMEDLLTMVGMGDSSATQQDESTIPTELSATRPEAPPPSIVASSPSSPAPAATPA
clc                 --------------------------------------------------------------------------------------------------EGQEAPPWESSSVAVP--SPATQTVPDVLEDMLAMVGMGNSSGTQQDEEATSHAADATPSEAPMPAMAAASPPSPVPPAAPS
PAGI-3_SG_          --------------------------------------------------------------------------------------------------DSCDSSPAASTTLTSS----TEPAADGVDDLLALLSAPDKGPPPPE--TVIQPLSTSSVSEPPTFAQESDAMQPPPMPSPT
PAGI-4_C_           --------------------------------------------------------------------------------------------------LRQPAPTIAKPSNDIQAIAKPSTDDQEETDDLYALLGNINSPPEELDTSHDSPAVSPANTRGEENLQQPLGTKEPIDCAPEA
pKLC102             --------------------------------------------------------------------------------------------------LRQPTPSVAKPSNEMRAIAKPSAEDQEETDDLYALLGNINSPPEELDTSHDSPAASSTNTRGEENLQQPLGTKEPTDCAPEA
Plasmid2_Ralme_     --------------------------------------------------------------------------------------------------EKAVNPSLAQADAPVPAPPPATSTQHADGKGNVSPAESAGLGSQAQVESPDATASDASAARNVVQLDGARSLRKELSADDPF
pMOL28              --------------------------------------------------------------------------------------------------EKAVNPSLAQADAPVPAPPPATSTQHADGKGNVSPAESAGLGSQAQVESPDATASDASAARNVVQLDGARSLRKELSADDPF
pHG1                --------------------------------------------------------------------------------------------------TGASTPPAQARTETAPVPETGTATPVEVEQPETTTEADPSSPAGLHQAGSDASAQHRASP-NAVQLDGARSLRKEAPAEDPF
pBVIE02             ---------------------------------------------------------------------------------------------------KVESAPPAAAPAPAPAEHVTADSSAAVAAAGGGAPADPEMPLASDSEGARPRPVPAPVLQGAPKGPDERQMALELPPAAAV
pMAQU02             --------------------------------------------------------------------------------------------------DPDPDPKAGEGHLDHQRTETHNNEYNQKTPKVASGPVTSKITTAHKEKGGAEPEEAKEKQSQVRAQPGSKAHGLTSEKAVEH
Rts1_IncT_          -------------------------------------------------------------------------------------------------AKGKAEAELSDKKSRSQNKKSEKAASPVAEKAVPEQTEDQGELFGIDNRYNDYPHVDTDEENDTYDLMEQYNNQTQEEPPLTL
pCAR1_IncP7_        ---------------------------------------------------------------------------------------------------EEEEAPQEPPASLEEMLLFLSPHSPAADPHQEETLSAAEAEISSDLSDADPQAGPGQGAQELLPRQEKISLNSIMGQGKRT
Plasmid1_Rhofe_     -------------------------------------------------------------------------------------------------DVVSVAPALAKEVQEIAILAPKSDLKAKVLESVDQETGEIAPVVASKPAAPEPPKTSAAAPAPASDVVQQVKPAVAAKRAPSA
Plasmid2_Polsp_     -----------------------------------------------------------------------------------------------------YGKAMVEAQAEQLTLAAARTVDAVNTPRAAGAAAEQPSALVADAPTTQVKVTTSAASPSANKESSAHKVPPALVVSTDS
Plasmid1_Polsp_     --------------------------------------------------------------------------------------------------EKKAKPAQANLEVDTPAVSTSPANATVDPETGELIASTEVRNDVSSTDGADLSADSAPVEPVAVTEVATAPIQPPALATAPL
chrom_Neigo_        ------------------------------------------------------------------------------------------------LVQENIRVEVKTETVDKPRAEVKETETDKAPKTIKSGTKASRKKLAGLFADNRKPIGKTAEPDAQIKTTETVTHQIDSEAVSLP
pPNAP01             -----------------------------------------------------------------------------------------------------LQTVPASTTTESAIVNTPKAKSISSNCENQKILQVVTLNVASEQRTNQININSLTDSKLKLENDQKLENKSVPGIPPKV
Plasmid1_Shesp_     ----------------------------------------------------------------------------------VEVSKEKTCIELLIEYLLTANNTLHRAKLDGDVLTLNASLFEEIDQQMRGLGFAEGAVLKDLMDNKLLIPDPKAPRKVIHVVNKQKFYQLKLADVLAK

                             910       920       930       940       950       960       970       980       990      1000      1010      1020      1030      1040      1050      1060      1070      1080
                    =========+=========+=========+=========+=========+=========+=========+=========+=========+=========+=========+=========+=========+=========+=========+=========+=========+=========+
R27_IncH1_          PATDPQSSDFAQLPRETALSVAPGDLDYSEGAIKPPAPDATGKETILTSPEPAEDVRETVAAVEKASHLSPALARLFAVS------------------------------THAEKKHEKTQEPSPVKEVKNPTSSTTVKAPISIEPP-------GAEEKEAVEEFTLLNDGEVTELEYVE
R478_IncHI2_        ATSSSVHNEKLLSEPSELPELNREQNADPQGTNERSMDVSVGQENSEPDTEGNCPPPAEVVYSQTEATATSVMASEEPALPPVLEESNGEHAPTDAKG---HHLSPALARLFAPTAPVEKQNPKRNRNKSSDKAEVQKPASPVSGHN-LNSKVFASTESDQNGEFSLISEGDVTELEFVE
R391_IncJ_          ISKR---------------LIALIDISKQGTEPAAAIQNEEALQRPSRTKTTN----------------------------------------------------------------AQAKEPATRAERKQKPIAPN----------------------------AKSSTDPKYVQRQQM
SXT                 ISKG---------------LTSLIDLPKQDAEPAAAIQNKEASPRPSRTETTN----------------------------------------------------------------AQAKEPAKRAERKQKPIAPN----------------------------ANSSTDPKHAQRQQM
pAsa4               IAAIKQAESLLGGYQDAFELVASPEVKKTQSKKNRGQRKTSRSESSGEDSHAASPERAQVEEKRVIADAQLIDVSVEPPSPPVDTTPPPDKVVPRKESRGKEKVDKPIQPAPTTKPTEGTQNLARLPPREQPKPESQ--QKQARVIDVG-----NIALP---GPDLPEELSPRSIVKEET
pIP1202_IncA/C_     VAAIKDAEASMGGYQDAFELVSPPGLDASKNKSAPKQQSRKKAQQQKPEVNAG---KASPEQK-----AKGKDSQPQPKEKKVDVTSPVE-----------EQQRKPVQ---------EKQNVARLPKREAQPVAPE--PKVEREKELG-----HVEVREREDPEVREFEPPKAKTNPKD
pAQU1               KAAIEQAEKDF----DPIREVSALNDKRQSGKKAEKAHSEKKVKSQKTNQSTPKKPKPAMDISALLPGEQTGGRSSENKRIAQQTAQGKKGTAAK------TQTEQHKNESENSASNEEKQNVSRLPKRQKPKTTVNKPPKETRPVSLDFDSDLELPMTKVVGQIDLEDEEPKKEQKAKE
PAGI-2_C_           ATTVQPS------GEHFMAWLKQGIASRRLIINDAKALVHTVSD---------------------------------------------------------------------------------------------------------------------------TAYLVSPGVFQRY
clc                 SATAQPS------GEHFMAWLKQGIVSRRLIINDAKALVHTVSD---------------------------------------------------------------------------------------------------------------------------TAYLVSPGVFQRY
PAGI-3_SG_          ENGGNAS------GEHFLAWLRHGIQSRKLIINDTKALVHTVAN---------------------------------------------------------------------------------------------------------------------------TAFLVSPGVFQRY
PAGI-4_C_           IEDVFMPSRSTDLGQGFVDWMKSGITARRLFINDTKALVHTVDG---------------------------------------------------------------------------------------------------------------------------TAMLVTPGIFKRY
pKLC102             VEDVFMPSRSTDLGQGFVGWMKSGIAARRLFINDTKALVHTVDG---------------------------------------------------------------------------------------------------------------------------TAMLVTPGIFKRY
Plasmid2_Ralme_     K------------------------------------ALAGSSN---------------------------------------------------------------------------------------------------------------------------LMREFFKALAQDV
pMOL28              K------------------------------------ALAGSSN---------------------------------------------------------------------------------------------------------------------------LMREFFKALAQDV
pHG1                K------------------------------------AIAGTSN---------------------------------------------------------------------------------------------------------------------------LMREFFKALSQDA
pBVIE02             NPAEIRR--------------EPGAAPATPAGERFDEILKGAPR---------------------------------------------------------------------------------------------------------------------------MISDFFAALRQDV
pMAQU02             WKTKREVQEDILRENDFLAWLLKGIARRQIRVNEPKAPVHILDG---------------------------------------------------------------------------------------------------------------------------HVALVTPAIFNLF
Rts1_IncT_          QERMARAAIQKQYEQDERNINQDGAPKESSWEKARAKIRQAQDITDGHAVTSDTYPHTESADVSAFIDDDHHFSAMFNDGPDEAKQILKRLVKERAG--------------------ELFADNYHLFVHKQNEQDEHDYTVLVNAGWVWKPFLEPMDNFQLYKSKPGFLLRRGLNEDIDY
pCAR1_IncP7_        PKTTPPAESKKQKDGEVGRVAEEVPCASSILPADVVSKLTAHECYLLEQQPDLAQKLITATQ--------------------------------------------------------DPENAALARNKVFVRLGKSSFLAEDIHPLVHAGWLWQDITVEGPALTRTLQSREGFMLTADY
Plasmid1_Rhofe_     KPGIP-------------------------TVPDVDDLVQETFDT-------------------------------------------------------------------------------------------------------------------------DMLLELGAEICREV
Plasmid2_Polsp_     VEVGGDS----------------SPHPGKRVPQDWLKRLKMKDDD-------------------------------------------------------------------------------------------------------------------------DTANALGMVIEQSL
Plasmid1_Polsp_     PGQSG----------------RIKEAAEVKFSDLVPEEIRKEIKT-------------------------------------------------------------------------------------------------------------------------TLTIELLGKVIKAW
chrom_Neigo_        ESESAQNGMRELERLRLREPTSSPRPVQVMDDSEGLDSLASGVKNMAELEAIVS---------------------------------------------------------------ERNLKKQSETESTEIVEDSVKAKHMEARDRGMQFLRWLADGLGDGSIAVNRSGATVHEIEQGM
pPNAP01             T--------------------------------------------------------------------------------------------------------------------------------------------------------------------------FWLRALISDEA
Plasmid1_Shesp_     PVGDMLTELQAKADERKARKQKNKALTTEVITEPNSLPTQESSIKDVIDGPKTEVPEQELSPLMPNEVPPESSCHSLMAEVPEQDLSSLMTDELP--------------------PLSEYAREMSLATPENELPWFTPEEQSMLTTDMEPTVPVAEAAIETAVEPATTSASFTEPAEQV
```

Fig. S1 (continued) Nonaka *et al.*

```
                                  1090      1100      1110      1120      1130      1140      1150      1160      1170      1180
                        =========+=========+=========+=========+=========+=========+=========+=========+=========+=========+=======
R27_IncH1_              IATMLHQILTKLSGSFKR--KRKNRFMVLTQNTFYITQSCIEKYG---TQLNAPDLFNQLPQYQVTSGAVVNTKCIAFNIPTLVAASDRAKVDIELIINKLKEVGNL
R478_IncHI2_            IALVLHQILSKMEVAFKR--KRKNRFMVSTPNTLYITQSCVEKFG---SQLEAQDLFNKLPQYLVNSGAVINTKCHAFNMPTLLAASDRAKVDIERIINNLKEAGNL
R391_IncJ_              VNFVKDLPILLTDGDYPD--VDHSADGIR----------------------VTIQTLRQVANAHGIPAGQLLRGISASDECQ--FDEGETVLFTAHAKR--------
SXT                     VSFVKDLPILLTDGDYPD--VDHSADGIR----------------------VTIQTLRQVANEHGIPAGQLLRGISASDQCQ--FDEGETVLFTAHAKR--------
pAsa4                   VDEALYLPP--KMTPEKA--IQMLKEMIKNRSGRWIVSAVLEEEG---FLTTSDKAFEVIAGEYTGISKHVLGGTLRRAQRRPLLKQRQGKLYLEVDKS--------
pIP1202_IncA/C_         INAEDFLPS--GVTPQKA--LQMLKDMIQKRSGRWIVTPVLEEDG---CLVTSDKAFDMIAGENIGISKHILCGMLSRAQRRPLLKKRQGKLYLEVNET--------
pAQU1                   ISDEDFLPPQSDIDAEEA--IRLLKEMIKNQKGRWIVGPVIKEDG---CLVTSDKALEKVTGECTNLSKSMLRALLASGQRRPRMTIRQGNLRLEVE----------
PAGI-2_C_               AQEHPQLAAIARQEKLEP--WQWAQKRFEKLAAHRKQASGLNIWT---CHVSGPRKSRQLHGYLLTQPGALFERVPPNNPYLCLFNEEAGREVSLAAK---------
clc                     AREHPQVGMLAKQESQQD--WQWVQKRFEKLQLHRKHINGLNVWA---CSVTGSRKSRQLHGYLLRDPLPLFGKVPPNNPYLSILQSI-------------------
PAGI-3_SG_              AQEHPQTAALAKQDQVAD--WQWVQKRFEKLQMHRKQDNGLNIWT---CEVTGPRKSRRLHGYLVSDPRHLFEEVPLNNPYLQLT----------------------
PAGI-4_C_               VQEHPEVEKLAQAKETAG--WKLVQRAFEKQGLHRKTSKNLNIWT---IKVSGPRKTKELKAYLLQDPKLIFPVQPLDNPSLTVITDAEGGVE--------------
pKLC102                 VQEHPELEKLAQAKETTG--WKLVQRAFEKQGLHRKTSKNLNIWT---IKVSGPRKTKELKAYLLQDPKLIFPVQPLDNPSLTVITDAEGDVE--------------
Plasmid2_Ralme_         ASGKAKVARIDGKVSISK--RSLGNFGLASDTLVEHLRKGKHLYK---VDGQNILLVDEVGRLIAPETAS-------------------------------------
pMOL28                  ASGKAKVARIDGKVSISK--RSLGNFGLASDTLVEHLRKGKHLYK---VDGQNILLVDEVGRLIAPETAS-------------------------------------
pHG1                    AAGKTKVTRVEGKVAISK--RSLGNYGIASETLIENLRKFKHLYK---IVGQDILLVNEVGDLILPASQAPEA----------------------------------
pBVIE02                 AGGKAKVRWAEKGLVVPK--RTIGGYGVASDTLIEHMRRRNLLLAN---DPTEVTLAPRIGELILERGA--------------------------------------
pMAQU02                 LDKNSLKKRLYEKRAGDKRVYTVLQRELEVLDIHQRGTDGQNIVT---VSVEGQRSKSELKVYLLNRACFPSLRAFAANPAIKIHL---------------------
Rts1_IncT_              LSGGTYLKSIYPVDPNKS--YCTDSALYRAIASYGILKQTFDNKD---VIAISGSAIRNMAKSLRIDTVTVRFLICYNFDSVSKRGSEHVLVDENIKPYMVDSHE--
pCAR1_IncP7_            SVIYCKLAELDWAIPHLSRLREADIPSLEQAVKSLIEKAVLEEIGGSPVLSLTTYMLTNVAEQFGITSTSLENAVFCFRDAVKVAKRRKIFVRPLYEELPKK-----
Plasmid1_Rhofe_         AAWRDLWNRGQSSQEFLRTTEGLAVSYAVVNGSAIQLSKIMEALKG-----AGYIEISNLNGTQRTLTPIPFPKKTELGLVLRTNFARKAGFILE------------
Plasmid2_Polsp_         AYRGDRVKVLDWGVAICVNWLTNTAGYDLADVLNPIERVGVIARN---PQEKGVALVSKVVFSDSPTPRMAFAVKLDFAAKVGMAIDAKV-----------------
Plasmid1_Polsp_         RERGEQSTTMRLTDNGAAISIDFLGTLMRSIPDWANEMASAGLIY-----APPDRPGLKVHKVAIPEGSKAKEAIVISRYGCKKLGL--------------------
chrom_Neigo_            LLVTPAVFRDYAGGVFNKSDTESLGPLTQKGFEALRLHARTKKSSLHRVLTTNGSNRRLFYCYLIPEHNIQHIIQPGSRPQNNTDIKLDESDLLVMEQK--------
pPNAP01                 MRLAFKFLPDQEMVDITG---AQLRFGVVAKEMFEILHSSGFVHS------KVGNLTVRLNKELTAVVQNEFKNLGVTHA---------------------------
Plasmid1_Shesp_         STLSQLKVATGNKSPKEKSKYPPELQVFIGWAMARAGKSGIDLRPG--VIRIQSFALTTYEQEVGLAKKEATSLKRTAGDNASIATDSNQVAWMVFETVGIEHE---
```

Fig. S1 (continued) Nonaka *et al.*

Table S1. List of MOB_H plasmids used for phylogenetic analysis of relaxase (TraI) [a]

Name of plasmid or Mobile element (ME)	Accession number of plasmid or ME	Bacterial source	Relaxase name	Accession no of relaxase	Size of relaxase (aa)	MOB_H Group	Experimental evidence for transfer	Reference
R478	BX664015	*Serratia marcescens*	hypothetical protein SMR0211 TraI	NP_941280	1050	H11	YES	Hedges et al., 1975
R27	AF250878	*Salmonella typhi*	TraI	NP_058333	1011	H11	YES	Maher & Taylor, 1993
Plasmid 1	CP000470	*Shewanella* sp. ANA-3	Relaxase	YP_863780	941	H11		
Rts1	AP004237	*Proteus vulgaris*	TraI	NP_640161	892	H12	YES	Terawaki, et al., 1967
pCAR1	AB088420	*Pseudomonas resinovorans*	TraI	NP_758664	900	H12	YES	Shintani et al., 2006
SXT	DQ180350	*Vibrio cholerae*	TraI	ABA87024	716	H12	YES	Waldor et al., 1996
R391	AY090559	*Providencia rettgeri*	TraI	AAM08003	716	H12	YES	Coetzee et al, 1972
pAQU1	AB571865	*Photobacterium damselae subsp. damselae*	TraI		1025	H12	YES	This study
pIP1202	CP000603	*Yersinia pestis* biovar Orientalis str. IP275	TraI	YP_001102157	992	H12	YES	Welch et al., 2007
pAsa4	CP000645	*Aeromonas salmonicida* subsp. *salmonicida* A449	Relaxase	YP_001144215	1006	H12		
clc genomic island	AJ617740	*Pseudomonas putida*	hypothetical protein	CAE92900	615	H2	YES	Mokross et al., 1990
PAGI-2(C)	AF440523	*Pseudomonas aeruginosa* strain C	conserved hypothetical protein	AAN62129	630	H2		
PAGI-3(SG)	AF440524	*Pseudomonas aeruginosa* strain SG17M	conserved hypothetical protein	AAN62266	600	H2		
pKLC102	AY257538	*Pseudomonas aeruginosa*	TraI	AAP22591	639	H2	YES	Qiu et al., 2006
PAGI-4(C)	AY258138	*Pseudomonas aeruginosa* strain C	conserved hypothetical protein	AAP94681	608	H2		
pMAQU02	CP000516	*Marinobacter aquaeolei* VT8	Relaxase	YP_957144	624	H2		
Chromosome	AY803022	*Neisseria gonorrhoeae*	TraI	AAW83058	850	H2		
Plasmid 1	CP000317	*Polaromonas* sp. JS666	hypothetical protein	YP_551982	493	H3		
Plasmid 1	CP000268	*Rhodoferax ferrireducens* T118	Relaxase	YP_515952	642	H3		

Plasmid 2	CP000318	*Polaromonas* sp. JS666	hypothetical protein	YP_552224	611	H3		
pPNAP01	CP000530	*Polaromonas naphthalenivorans* CJ2	hypothetical protein	YP_973353	408	H3		
pBVIE02	CP000618	*Burkholderia vietnamiensis* G4	hypothetical protein	YP_001109781	441	H3		
pHG1	AY305378	*Ralstonia eutropha* H16	hypothetical protein	NP_943000	418	H3	YES	Friedrich et al., 1981
Plasmid 2	NC_007972	*Ralstonia metallidurans* CH34	hypothetical protein	YP_582117	413	H3		
pMOL28	X90708	*Ralstonia metallidurans* CH34	TraI	YP_161633	359	H3	YES	Mergeay et al., 1985

a This list is referred reference no.15.
